# Effect of the Order-Disorder Transition on the Seebeck Coefficient of Nanostructured Thermoelectric Cu_2_ZnSnS_4_

**DOI:** 10.3390/nano9050762

**Published:** 2019-05-17

**Authors:** Eleonora Isotta, Carlo Fanciulli, Nicola M. Pugno, Paolo Scardi

**Affiliations:** 1 Department of Civil, Environmental and Mechanical Engineering, University of Trento, via Mesiano 77, 38123 Trento, Italy; eleonora.isotta@unitn.it (E.I.); nicola.pugno@unitn.it (N.M.P.); 2 Laboratory of Bio-Inspired and Graphene Nanomechanics, Department of Civil, Environmental and Mechanical Engineering, University of Trento, via Mesiano 77, 38123 Trento, Italy; 3 National Research Council of Italy-Institute of Condensed Matter Chemistry and Technologies for Energy (CNR-ICMATE), Lecco Unit, via Previati 1/E, 23900 Lecco, Italy; carlo.fanciulli@cnr.it; 4 Ket-Lab, Edoardo Amaldi Foundation, Via del Politecnico snc, 00133 Rome, Italy; 5 School of Engineering and Materials Science, Queen Mary University of London, Mile End Road, London E1 4NS, UK

**Keywords:** order-disorder transition, Seebeck coefficient, kesterite, CZTS, nanostructured materials, thermoelectric materials, crystal structure symmetry

## Abstract

Bulk samples of kesterite (Cu_2_ZnSnS_4_, CZTS) were produced by cold-pressing and sintering of CZTS powders obtained via reactive ball-milling. An increase in the Seebeck coefficient of more than 100 *μ*V/K, almost doubling the expected value, is noticed around a temperature of 260 °C. As pointed out by thermal analyses, this is due to a second order transition of kesterite from an ordered *I-4* to a disordered *I-42m* crystal structure. Conversely to what happens for solar cell materials, where the transition is considered to be detrimental for the performance, it appears to be beneficial for the thermoelectric Seebeck coefficient, suggesting that higher crystal symmetry and cation-disorder due to the transition lead to thermopower enhancement.

## 1. Introduction

**Figure 1:**
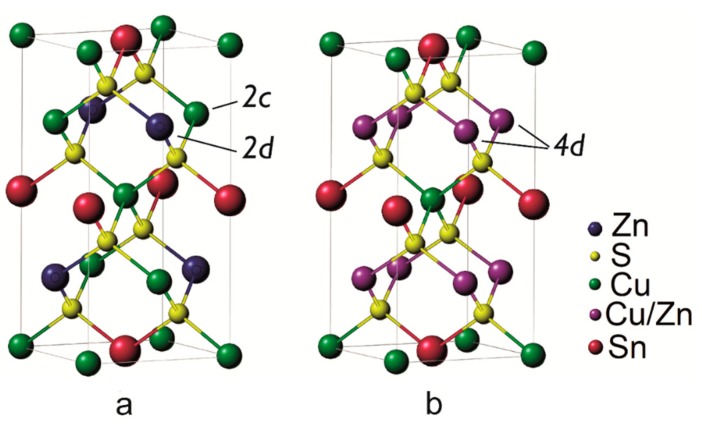
Ordered *I-4* (**a**) and disordered *I-42m* (**b**) crystal structures of kesterite. In the ordered state Cu and Zn occupy respectively *2c* and *2d* Wyckoff positions while above the transition temperature, they appear as randomly distributed in the planes of *4d* Wyckoff positions [14,15,26].

Thermoelectric materials can operate the conversion of a heat gradient into a voltage drop through the Seebeck effect [1,2]. The performance is usually expressed through the figure of merit *zT = S*^2^*T*/(*ρk*), where *S, ρ, T*, and *k* are, respectively, the Seebeck coefficient, electrical resistivity, absolute temperature, and thermal conductivity [3] although recently the thermoelectric quality factor *β* has been proposed, as it draws attention to the most fundamental material parameters to be improved (*β* ∝ *N_v_*/(m*_I_ k_L_), with *N_v_*, band degeneracy, m*_I_, inertial effective mass along the conduction direction, and *k_L_*, lattice thermal conductivity) [4,5]. Kesterite (Cu_2_ZnSnS_4_, CZTS) is a direct band gap p-type semiconductor that has recently been studied as a thermoelectric material [6,7,8,9,10]. The interest arises from the low cost, easy availability and non-toxicity, in addition to its low thermal conductivity (from 2.95 W/(m K) at 300 K to 0.97 W/(m K) at 700 K [7]). The reported values of figure of merit *zT* for kesterite remain quite low, ranging from 4.6 × 10^−5^ at 300 K to 0.026 at 700 K [7]. For this reason several studies have focused on doping to increase the electrical conductivity [6,7], without considering, until now, the importance of structural aspects and the role of phase transformations. Kesterite is also an object of intense research for the realization of absorbing layers in thin-film solar cells, valued for the high absorbance and nearly optimal band gap (~1.5 eV), which make it a candidate for the substitution of rare and potentially toxic metal chalcogenides [11,12,13]. In its usual form, kesterite has a tetragonal crystal structure belonging to space group *I-4*. Quite recently, it has been reported that a reversible second-order phase transition occurs at a critical temperature of 260 ± 10 °C [14,15]. This phase transition appears to involve the Cu-Zn planes in the crystal structure. As depicted in Figure 1, below the critical temperature Cu and Zn are in their ordered configuration, occupying respectively the *2c* and *2d* Wyckoff positions. Above the critical temperature, Cu and Zn are randomly distributed in the planes, leading to a transition to a *I-42m* crystal structure where both cations occupy *4d* Wyckoff positions. This order-disorder transition has been linked to the low formation energy of the Cu-Zn antisite defects [16]. At room temperature, kesterite has been reported to show a higher order degree, although this is primarily related to the thermal history of the sample and to its stoichiometry [17], and complete order is theoretically possible only at 0 K [15]. In photovoltaic applications, disorder has been reported to mainly cause a decrease in the bandgap energy *Eg*, leading to a lower open circuit voltage [15], but also a higher short-circuit current density [18,19]. Since Zn^+2^ and Cu^+1^ are isoelectronic, it is difficult to observe and quantify disorder with conventional X-Ray Diffraction (XRD), where the phase transition is just noticed as a smooth change of cell parameter with the temperature [20,21]. Other techniques, however, proved more effective such as solid-state nuclear magnetic resonance [22], UV-Vis spectroscopy for bandgap measurement [18,23], Raman spectroscopy [15,22,24], neutron scattering [21], and resonant XRD [25]. In this work we show how to effectively observe and study the order-disorder transition with the measurement of Seebeck coefficient, and we confirm the results with the aid of accurate thermal analyses.

Nanostructured kesterite is usually produced by chemical synthesis [27,28]. This approach typically requires a thermal treatment well above the critical temperature, and a slow cooling down to obtain, to the extent possible, defect-free and ordered crystals, deemed appropriate for the performance of thin film solar cells [14]. However, the effect of a production route of CZTS powders based on reactive milling at room temperature of precursors such as elementary (Cu, Zn, Sn) metals and sulfur is not well known. This is the approach followed in the present work to obtain a nanostructured material with finely dispersed and intrinsically disordered crystalline domains, designed to preserve the nanostructure after sintering and in operating conditions. Furthermore, we show the effect of the order-disorder transition, which is known for photovoltaic kesterite, but has not yet been investigated as regards the effect on thermoelectric properties.

## 2. Materials and Methods

Kesterite powders have been produced from stoichiometric precursors (Cu powder, <75 μm, 99%; Zn powder, purum, 99%; Sn powder, puriss, 99%; S flakes, purum, 99.5%; all by SigmaAldrich (Saint Louis, MO, USA) via reactive ball-milling with brass balls and vials in a planetary mill (Fritsch P4 Pulverisette 4, Idar-Oberstein, Germany) adding 150 μL of ethanol (99.8%, SigmaAldrich) as lubricant [29]. Milling has been performed for 60 min with jar rotation *ω* = −540 rpm and main disk revolution Ω = 300 rpm [30]. Powders have been cold-pressed with a manual pressing machine and a pressure of 25.5 MPa to obtain disks with diameter around 20 mm and thickness around 1.5 mm. The final disks have then been sintered in a tubular oven in Ar flux for 60 min at 300 °C (heating rate r = 20 K/min) followed by 20 min at 560 °C (r = 20 K/min up to 520 °C and 10 K/min from 520 to 560 °C) [31]. Some of the disks have been naturally cooled down to room temperature inside the furnace atmosphere, for a total cooling time of 8 h, others have been quenched in air to room temperature. XRD (Rigaku PMG, Tokyo, Japan, Cu K*α* radiation), Trasmission Electron Microscopy (TEM) imaging, Selected Area Electron Diffraction (SAED) and high-magnification Energy Dispersive X-ray Analysis (EDXS) (HR-S/TEM ThermoFischer TALOS 200 s, Thermo Fischer Scientific, Waltham, MA, USA) have been used to characterize the samples. Thermal analyses and specific heat measurements have been performed with a Thermal Analysis Q100 DSC instrument (TA Instruments, New Castle, DE, USA). This instrument allows a modulated mode (MDSC) for calorimetric analyses that is useful for studying materials with low thermal conductivity. The modulated mode has been used in two ways. In isothermal measurements, the temperature modulation has been applied to the thermal equilibrated sample to determine the specific heat as a function of temperature. In ramp mode, the temperature modulation has been applied to the imposed ramp rate (2 K/min), allowing the identification of the reversible and non-reversible contributions to the heat flow signal from the sample. In all cases, the applied modulation has a period t = 120 s, and semi-amplitude A = 0.5 °C. Absolute Seebeck coefficient (platinum standard) has been measured in a 4-contact configuration with Linseis LZT-Meter (Linseis Messgeraete GmbH, Selb, Germany) from 50 °C to 450 °C, with step of 10 °C, a heating rate of 10 K/min and an effective temperature gradient of approximately 10 °C.

## 3. Results and Discussion

**Figure 2:**
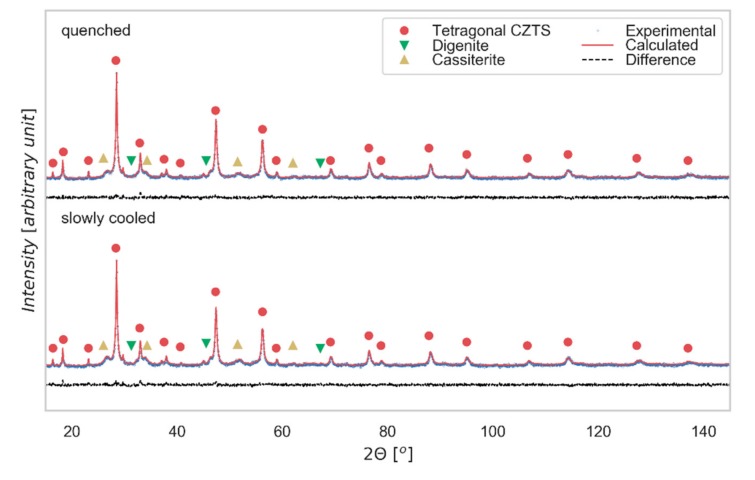
X-Ray Diffraction patterns. XRD data Rietveld refinement (performed with TOPAS 7) and phase identification for the slowly cooled and quenched samples. The difference between experimental data (blue) and model (red) is shown below (residual, black line).

**Figure 3:**
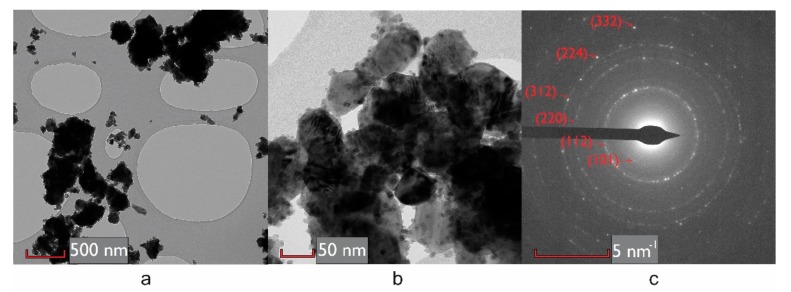
TEM and SAED images: (**a**,**b**) TEM images of slowly cooled sample ground to powder; (**c**) SAED pattern of the area shown in Figure 3a, Miller indices of scattering planes corresponding to a tetragonal crystal structure are indicated.

XRD data for both slowly cooled and quenched samples are shown in Figure 2, together with result of a modeling made with TOPAS 7 [32], whereas TEM and SAED images for the slowly cooled tablet are visible in Figure 3.

The XRD pattern shows the expected reflections of tetragonal kesterite including the distinctive weak peaks at *2θ*~18°, 23°, 38°, which reveal the main difference with respect to the cubic sphalerite-type structure [31]. The same indexing is confirmed by SAED (Figure 3c), where it is possible to notice the diffraction ring corresponding to the (101) plane, distinctive to the tetragonal structure. For both cooling processes, kesterite is the main phase. As expected for the isoelectronic character of Cu^+1^ and Zn^+2^, XRD cannot capture any relevant difference in the pattern of the two samples [15]. Additional peaks refer to secondary phases, identified by a search-match procedure supported by the PDF-4+ database (ICDD, 2019 [33]), and then quantified by the modeling of the XRD patterns as cassiterite SnO_2_ (weight fraction 12(1) % for slowly cooled sample and 9(1) % for quenched sample) and digenite Cu_7,2_S_4_ (weight fraction 5(1) % in both samples). The last phase might have a positive role, as it has been observed that its partially-metallic nature contributes to improving the electrical conductivity of CZTS thin films [8].

TEM images (Figure 3) show kesterite in grains and agglomerates ranging from about 10 nm to over 300 nm. Cassiterite, identified by EDXS, appears in small nucleated domains of around 10 nm preferentially located at grain boundaries of CZTS (Figure 3b). EDXS on single grains shows stoichiometry fluctuations from grain to grain of the kesterite phase. From these observations we noticed a tendency towards bimodality in the size distribution of kesterite: two fractions are mainly identified, a bigger one, in the range of hundreds of nanometers and a smaller one, in the range of tens of nanometers (see Figure 3a). This is consequence of the mild sintering process, designed to preserve the nanostructure in bulk samples, made for short time at 560 °C (833 K), well below the melting point of 1260 K [34]. Moreover, the high dispersion of domain sizes in the starting powder made by ball milling causes differences in grain aggregation and packing during the preparation of the green. In fact, the density of the starting pallets after cold pressing was 3.4 g/cm^3^ (geometrically measured on disk samples), 75% of the theoretical density reported for CZTS (4.56 g/cm^3^ [35]). Density fluctuations inevitably lead to differences in the growth rate of grains, resulting in the final inhomogeneous grain size distribution. Based on the observed bimodality, we used two different-size kesterite fractions to model XRD data. A TOPAS macro based on Whole Powder Pattern Modeling (WPPM) (Scardi, 2002 [36]) has been used to refine the domain size in terms of lognormal distributions of spherical domains [37]. For both samples, the estimated mean domain sizes are 20(2) nm for the smaller fraction (45(2)% of total CZTS) and 180(15) nm for the bigger fraction (55(2)%), matching the high dispersion of dimension that was experimentally observed with TEM analyses. The micro strain is quantified as 0.0004(1) and 0.0017(2), respectively for larger and smaller fractions. We believe that this should be considered, at least partially, as an effect of the stoichiometry fluctuation, confirmed by EDXS, rather than strain. In fact, fluctuations in the composition may cause the cell parameter to slightly vary, thus having multiple peak positions for the same reflection that lead to strain-like peak-broadening [38].

**Figure 4:**
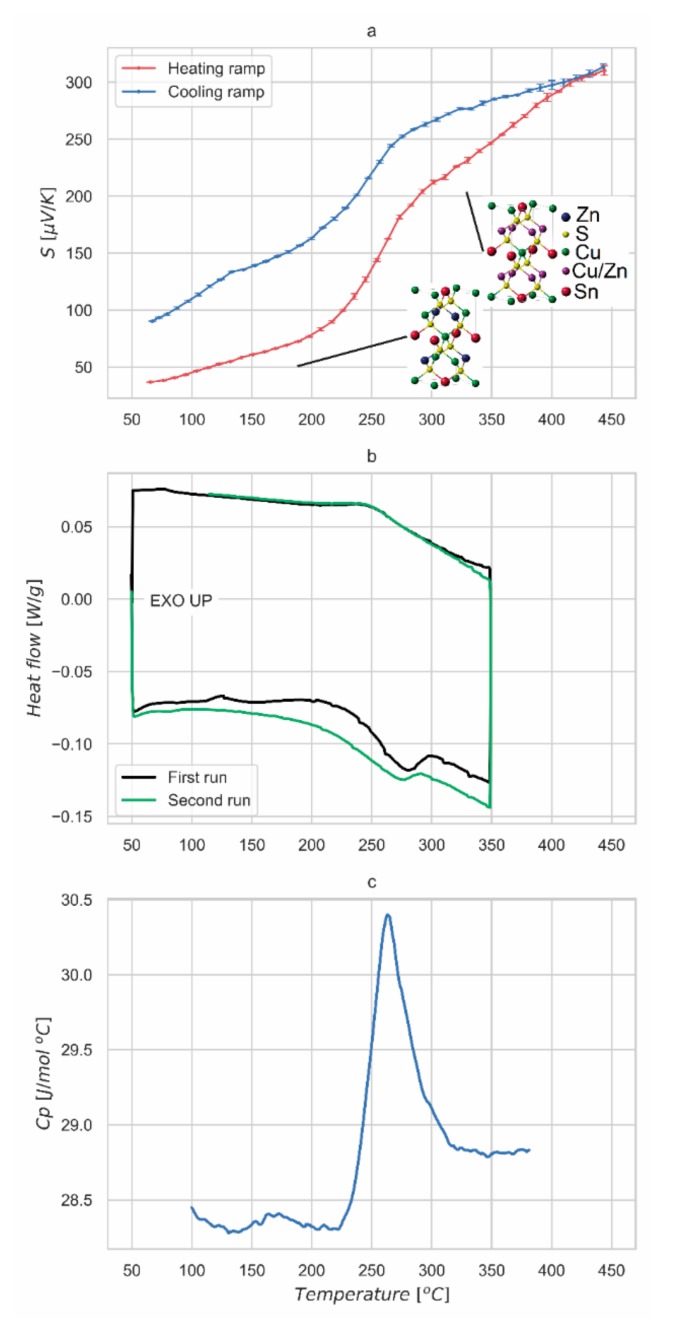
Seebeck measurements and thermal analyses. Absolute Seebeck coefficient measured for the slowly cooled kesterite sample in heating and cooling ramp, with insets representing the relevant crystal structures. (**a**). Thermal analysis (DSC) curve measured for the slowly cooled sample. First and second runs are displayed in both heating and cooling ramp. (**b**). A second-order phase transition is clearly visible, especially in the trend of the constant-pressure heat capacity (*Cp*), in (**c**).

Figure 4a shows the absolute Seebeck coefficient measured on the slowly cooled sample on heating and cooling temperature ramp. Values are positive, confirming kesterite p-type behavior and range between 40 μV/K at 50 °C and 315 μV/K at 450 °C. In this temperature range, it is interesting to note the marked increase of Seebeck coefficient occurring between 210 °C and 290 °C, where values pass from around 80 μV/K to over 200 μV/K. We believe this is related to the order-disorder phase transition in kesterite. The thermal treatment of the samples was performed with a peak temperature of 560 °C, well above the reported critical temperature of the order-disorder transition. Following a slow cooling to room temperature, Cu and Zn cations set their positions to *2c* and *2d* Wyckoff sites of *I-4* structure, respectively, leading to a partially-ordered kesterite. The extent of the ordering process critically depends on this cooling step, and on the stoichiometry of the CZTS phase. In fact, the capability of the cations to change their position is strictly related to the thermal energy of the system and a gradual temperature reduction allows the ordered arrangement of atoms on the Cu and Zn planes. The dependence of the ordering on thermal history could explain why some authors did not observe the phase transition [6,7], whereas others did (e.g., see Devi et al., 2019; Kumar, Ansari and Khare, 2018) [8,10], although they did not comment on it.

During Seebeck measurements, on heating phase, Cu and Zn form an increasing number of antisites, which tend to cluster in (Cu_Zn_ + Zn_Cu_) defect pairs until complete disorder is reached at the critical temperature. It is important to stress that the disordered kesterite has a different atom arrangement from stannite, although the *I-42m* space group is the same. On cooling, as already pointed out, the *I-4* ordered structure is partially recovered, to an extent depending on the decreasing temperature ramp.

From the observed trend of Seebeck coefficient, we can assess the reversible character of the transition, fully developed around 260 °C in the heating ramp: this is consistent with the order-disorder critical temperature reported by Scragg et al. [15]. The thermal hysteresis observed on the cooling ramp, lowering the critical temperature of about 20 °C, is probably related to the thermal inertia of the sample. This feature, associated to a decreasing ramp faster than the natural cooling cycle performed after sintering, can explain the difference in Seebeck coefficient observed at 50 °C between heating and cooling measurements.

It is interesting to notice how powerful the measurement of Seebeck coefficient is as a tool to study the order-disorder transition: it gives an indication of the transition temperature and of its reversible character in an easier and clearer way with respect to traditional structural characterization techniques, which are much more complex and less effective in studying this second order transition.

Thermal analysis, visible in Figure 4b, confirms the presence of a reversible transition. The transition has been observed between 240 °C and 250 °C on heating, while a low hysteresis can be observed in the reversal process during the cooling branch of the curve. The broadening of the transformation can be partially explained with the presence of secondary phases in the material. This could also explain the curve evolution between the first and the second run, probably associated with a material stabilization with thermal cycling. From the peak-shape it is confirmed to be a Landau second-order transition, matching what was previously reported for the order-disorder transition in kesterite [15].

**Figure 5:**
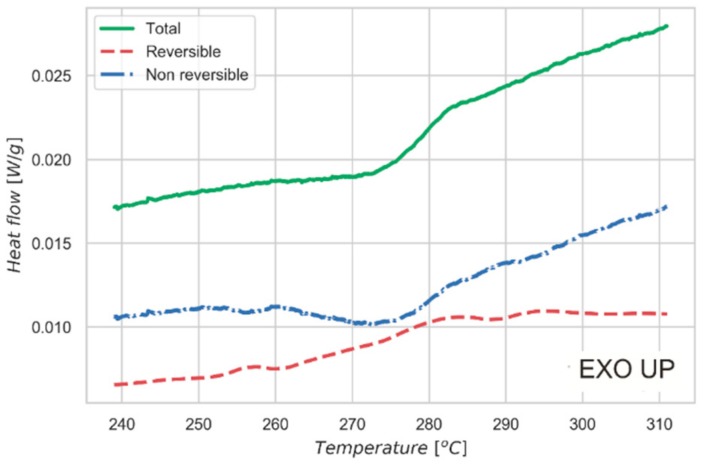
Modulated mode thermal analysis measurement (MDSC). MDSC is performed across the critical temperature to study the character of the order-disorder transition. MDSC allows us to separate the reversible (red) and the non-reversible (blue) contributions to the total heat flow in the sample (green). Ramp 2 K/min, modulation period 120 s, and semi-amplitude 0.5 °C.

Figure 5 shows the data collected by MDSC. The experiment allows us to separate the heat flow curve into two different contributions. One is the step change in heat capacity of the sample due to the order/disorder transition. This effect is reported in the reversible heat flow signal. The second contribution is associated with the enthalpy recovery of the system: this phenomenon is related to the kinetic of the measurement and depends mainly on the thermal diffusivity of the material and on the specimen size and shape. Such a contribution is visible as a peak in the non-reversible curve reported in Figure 5. This behavior is further proof of the nature of the transition observed in the material, which is also confirmed by the constant-pressure heat capacity (*Cp*) measurement over the same temperature interval. As shown in Figure 4c, a peak of *Cp* is observed at the order-disorder transition, with a shape characteristic of higher order phase transformations [39]. *Cp* of the high temperature (disordered) phase is higher than for the low temperature (ordered or partially-ordered) phase, demonstrating an increase in vibrational modes in the disordered phase.

**Figure 6:**
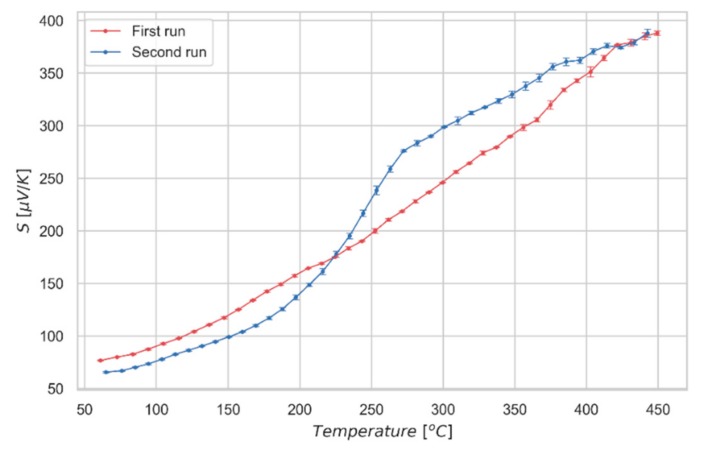
Absolute Seebeck coefficient measured for the quenched kesterite sample. First and second runs of the measurement are shown.

To validate these observations, we measured the absolute Seebeck coefficient of a quenched sample. Quenching was performed from 560 °C, well above the critical temperature, by sudden extraction of the sample from the treatment oven. Due to the fast cooling, we expect the material to be in a fully-disordered state after the treatment. The first run of the measurement of the quenched specimen (Figure 6) shows an approximately linear trend, whereas a second run, made after letting the sample to naturally cool down from the previous measurement, shows the characteristic transition shape at around 250 °C. These results support the idea of a fully-disordered state for the as quenched material (first run), displaying a linear Seebeck coefficient; whereas, upon a slower cooling, kesterite partially recovers the order, displaying a sharp increase of Seebeck value during the second run, similar to the one already observed for the partially-ordered samples of Figure 4a. Further measurements of Seebeck coefficient are reported in the Supplementary Materials to show reproducibility of the analysis and the limited variability of the results.

It is clear how the transition strongly affects the Seebeck of the material. In fact, in all reported cases the high-temperature Seebeck far exceeds that of the low-temperature structure. A reason for this behavior could be found in the increasing structural symmetry of the system associated with the order-disorder transition. As reported in the literature for a parental compound, the chalcopyrite Cu_2_MGeSe_4_, by Zeier et al. [40] and by Zhang et al. [41], the symmetry increase of the crystal structure leads to a loss of band divergence in the system, which in turn raises the density-of-states effective mass, $m_{DOS}^{*}$, of the carriers due to the increased degeneracy of the electronic bands. In the cited paper by Zeier et al. the symmetry of the system has been controlled by introducing different elements in the cell in order to obtain a pseudo-cubic structure. Here, kesterite displays a similar behavior associated with the second-order transition. The Cu-Zn arrangement above the transition increases the symmetry due to the large number of (Cu_Zn_ + Zn_Cu_) defects promoted by the thermal energy. In fact, above the order-disorder transition, the ordered atomic arrangement, having Cu atoms in *2c* (0,1/2,1/4) positions and Zn in *2d* (0,1/2,3/4) ones, leaves the place to a statistically arranged distribution of Cu and Zn on the *4d* (0,1/2,1/4) positions [21]. Such disorder involves an increase in the symmetry of the system, which can result in a narrowing of the gaps between the different bands at *Γ* point. This effect could lead to a band degeneracy due to the removal of the crystal field contribution, *Δ_CF_*, to the band spacing. As reported in the literature [42], for CZTS the spin-orbit coupling doesn’t affect the band degeneracy due to the light weight of S atoms.

To demonstrate the degeneracy mechanism, however, it is necessary to check in detail the band structures of the ordered and disordered states of Cu_2_ZnSnS_4_. Apparently, this information, so far, is only available for the ordered kesterite, close to our low temperature structure, and stannite [43,44], with same crystal structure of our disordered kesterite, but corresponding to low temperature and different atom arrangement. To the best of our knowledge, no information is reported in the literature on the band structure of disordered kesterite, possibly including the effects of stoichiometry fluctuations and temperature, in the range explored by our measurements of Seebeck coefficient.

The influence of the structural state of the material on its band structure, could also explain the difference observed in the Seebeck measurement performed on the rapidly cooled sample (Figure 6). In fact, the first cycle not only differs in terms of the absence of transition evidence, but also displays unexpected absolute values: considering the disordered state at low temperature kept by the quench from high temperature, values of Seebeck coefficient in the first cycle should be higher, matching the linear trend observed above the transition during the second run. However, the quenching process promotes not only a disordered structure, but also structural distortions; indeed, quenched samples tend to deform, as a clear consequence of residual stresses. The thermal cycle performed by the sample during the measurement, followed by a slow cooling at room temperature, allows the structure to recover from all the effects induced by the fast cooling. As a consequence of structural stresses, the measured Seebeck values, despite being higher in the first run, are not as high as they should for the lack of full recovery of the system. The convergence of the Seebeck values can only be observed at high temperature, where residual stress is completely relaxed.

The present work was not aimed at showing a high-performance thermoelectric material, but rather to show the structural information provided by a simple measurement of Seebeck coefficient with the support of thermal analyses. Moreover, the order-disorder transition of kesterite known in photovoltaics was presented from a different perspective, as a proof-of-concept of the possibility to achieve a higher Seebeck coefficient with increased disorder and crystal symmetry. It is however possible to estimate a figure of merit zT at 400 °C for our samples, ranging from 0.025 to 0.04, comparable with the literature values for thermoelectric kesterite at the same temperature [6,7]. For this estimate we used the specific heat of Figure 4 and the published data of electrical resistivity and thermal diffusivity [31]. The latter were recently measured on a kesterite sample obtained with the same process, apart from a faster cooling down process after the thermal treatment; the zT value should be unaffected in any case by the cooling velocity, since the sample at 400 °C is in a complete disordered state. More extensive measurements and developments to improve electrical resistivity and thermal diffusivity of the studied system are part of the ongoing research work.

## 4. Conclusions

The present work demonstrates the effect of the order-disorder transition of kesterite on the Seebeck coefficient. This transition, known as a detrimental effect for the performance of solar cell materials, turns out to positively affect the thermopower. *Cp* measurement and thermal analyses, including modulated mode measurements (MDSC) to decouple the reversible and non-reversible components of heat flow, confirm what was previously reported in the literature about the character of the transition [14]: it is a second-order Landau phase transition, completely developed at 260 °C and fully reversible upon slow cooling. The Seebeck curve presents a sharp increase of more than 100 μV/K at the transition temperature, from around 80 μV/K at 210 °C to over 200 μV/K at 290 °C. The effect of Cu-Zn disorder has been further assessed by measuring the Seebeck coefficient of a quenched sample (in disordered state, *I-42m* crystal structure) and observing that it follows a linear trend, while after slow cooling (partially recovers ordered *I-4* crystal structure) displays a sharp increase due to the transition. Measuring the Seebeck coefficient also proved to be a simple and efficient way to observe the transition, which is difficult to spot using traditional structural characterization techniques [20,21]. What shown in this paper leads us to think that the order-disorder transition of kesterite and, in a wider sense, the evolution towards a more symmetrical crystalline structure and the cationic disorder, can lead to an increase in band degeneration and to the consequent improvement of the Seebeck coefficient. This indicates some promising study directions for further improvement of thermoelectric performance.

## Supplementary Materials

The following are available online at https://www.mdpi.com/2079-4991/9/5/762/s1.
