# Supplementary material for: Effect of the Order-Disorder Transition on the Seebeck Coefficient of Nanostructured Thermoelectric Cu2ZnSnS4
Source: Nanomaterials (Basel). 2019 May 17;9(5):762. doi: 10.3390/nano9050762 (PMC6567239; doi:10.3390/nano9050762)
Supplement: Supplementary file 1 [file nanomaterials-09-00762-s001.pdf]

Supplementary material

Effect of the Order-Disorder Transition on the Seebeck Coefficient of Nanostructured Thermoelectric Cu_2ZnSnS_4

Eleonora Isotta [1,2], Carlo Fanciulli [3], Nicola M. Pugno [1,2,4,5] and Paolo Scardi [1,*]

Figures S1 and S2 show additional measurements of the absolute Seebeck coefficient for second runs on the samples described in the main text of the article and for some additional samples, in order to assess reproducibility of the analysis and variability of results.

The second run performed on the same sample presented in Figure 4 of the main text is presented in Figure S1 (yellow line), together with the measurement of another sample obtained with the same processing route (green line). The measurement on the same sample is strictly reproducible, whereas the variability of the result between different samples is within 10%; in all cases it is confirmed the trend with the temperature, showing a steep increase of Seebeck coefficient for the order-disorder transition, occurring at approximately the same temperature. The high reproducibility of the transition temperature observed in the different samples supports the association of this characteristic behavior to the material itself and not to a peculiar character of the sample. The sample-related characteristics of the curves can be measured in the discrepancy in the absolute values, while the characteristics of the curves in terms of material thermo-physical properties remain practically unchanged. The Seebeck coefficient in heating and cooling ramps for the sample presented in the main text is reported for a sake of comparison (red line: on heating; blue line: on cooling).

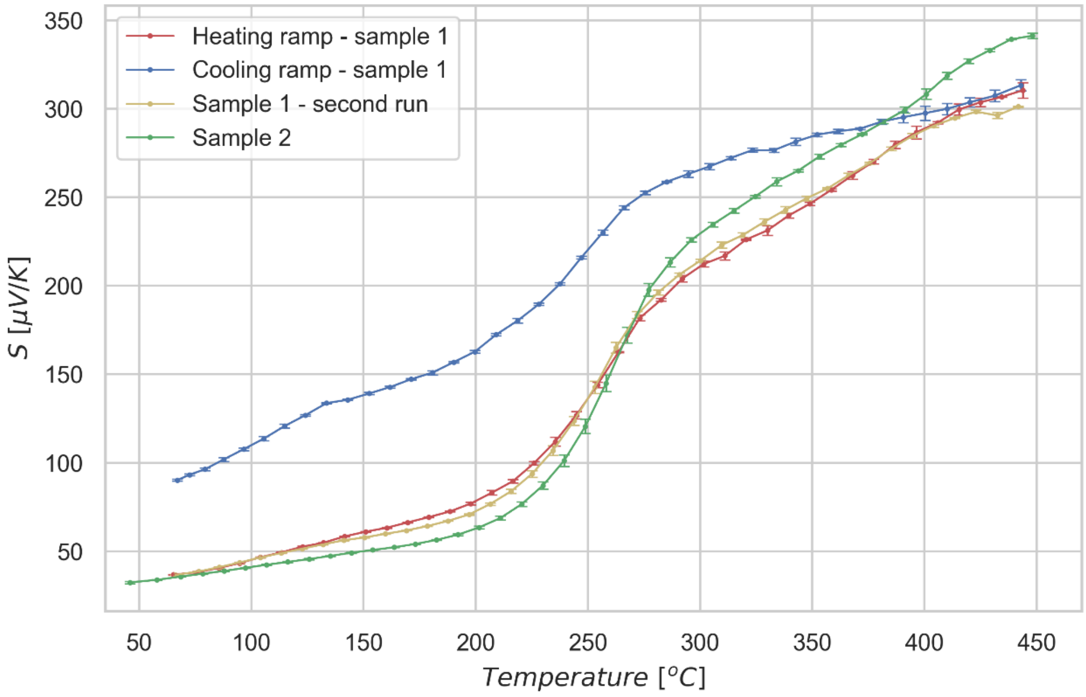

Figure S1. Absolute Seebeck coefficient additional measurements: for the same sample reported in the main text (sample 1) and for an additional sample (sample 2), prepared with the same procedure as sample 1.

The first and second runs of the Seebeck coefficient measurement for the quenched sample presented in Figure 6 of the main text (here sample 3) are shown again in Figure S2. On the same figure, the first and second

run of the Seebeck coefficient for another quenched sample (sample 4). This sample has been obtained with the same processing route described in the main text but different quenching: instead of being quenched from 560°C (as with sample 3), sample 4 was slowly cooled from 560°C to 450°C, and then quenched to ambient temperature. The first run of sample 4 displays a mild increase at the order-disorder transition temperature, similarly but not identically to the behavior of sample 3. This is likely due to the different, less effective quenching process, from a lower starting temperature, which caused the sample to partly recover the ordered CZTS structure, thus mildly displaying the order-disorder transition. Upon the successive slow cooling sample 4 recovered a larger, more complete ordered structure, thus presenting a sharper increase in the following run (Second run – sample 4). Variability of results between the samples is within 15%.

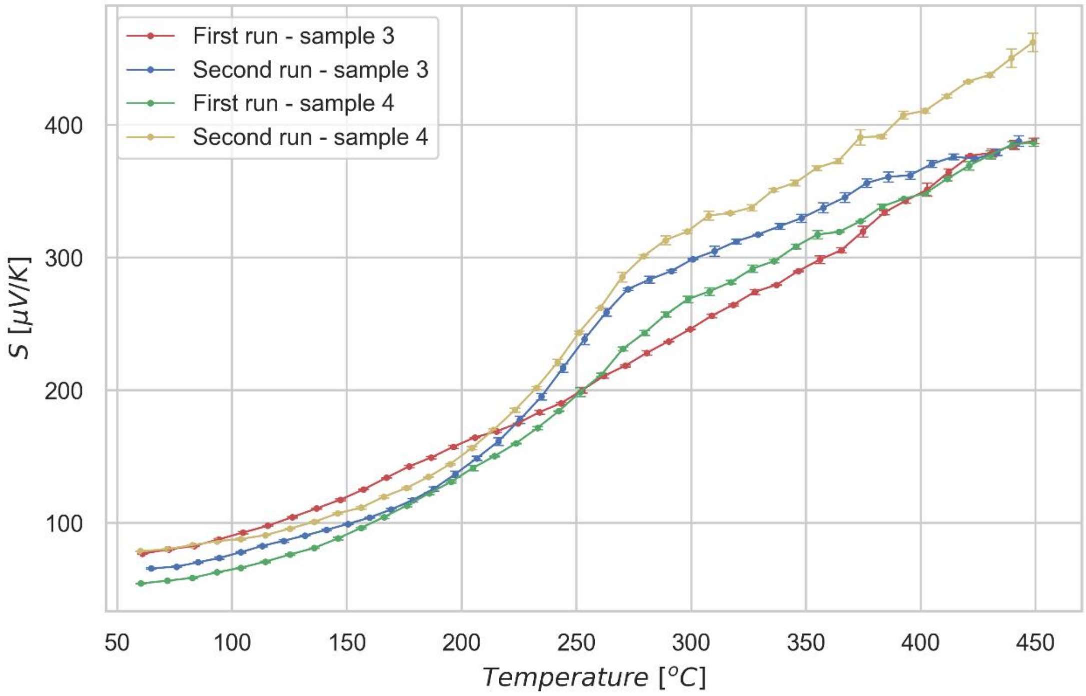

Figure S2. Absolute Seebeck coefficient additional measurements: for the same quenched sample reported in the main text (sample 3) and for an additional quenched sample (sample 4).
